# Supplementary material for: Wind loads and competition for light sculpt trees into self-similar structures
Source: Nat Commun. 2017 Oct 18;8:1014. doi: 10.1038/s41467-017-00995-6 (PMC5715076; doi:10.1038/s41467-017-00995-6)
Supplement: Supplementary file 1 — Supplementary Information [file 41467_2017_995_MOESM1_ESM.pdf]

SUPPLEMENTARY INFORMATION

SUPPLEMENTARY METHODS

Parametric analysis on the self-similar ratios

In the present paper, the fittest species is identified through an evolutionary tournament method. This approach is inherently stochastic, computationally costly (about 3,000 CPU hours for a single tournament), and this drastically constrains our capacity to conduct a detailed sensitivity analysis. To circumvent this difficulty, we have run a parametric analysis instead to assess how self-similar ratios depend on the model parameters.

In this parametric analysis, we simulate during 200 yrs the growth of an isolated tree belonging to the fittest species. The reference case corresponds to values given in Table 1 and we compare this case to other runs with different values of the Cauchy number C_Y, the maintenance thickness e, the volume of photosynthates produced $V_{\text{prod.}}$, and the foliage transparency $\alpha_{\text{fol.}}$ (Supplementary Fig. 12).

It appears that trees grow to a larger size when C_Y or e is decreased, or when $V_{\text{prod.}}$ is increased (Supplementary Fig. 12b–g). These size variations can be explained with simple arguments: a lower Cauchy number or a lower maintenance thickness corresponds to fewer photosynthates spent on secondary growth allowing the tree to grow larger, and the same is achieved if a larger amount of photosynthates is produced by foliages, as expected. The remarkable property of these simulations is that, whereas tree size varies over more than an order of magnitude, the branching and length ratios do not vary significantly. For the different values of these parameters, the branching ratio is $3.4 \leq R_n \leq 3.7$, the length ratio is $1.6 \leq R_l \leq 1.8$, and the fractal dimension is $2.2 \leq D \leq 2.5$ (Supplementary Fig. 12b–g). This shows again that the self-similar properties do not significantly depend on the values of these model parameters.

The same is not true when studying the effect of the foliage optical transparency $\alpha_{\text{fol.}}$. As shown in Supplementary Fig. 12h–i, the fractal dimension is lower ($D \approx 2.1$) when foliages are fully opaque, and larger ($D \approx 2.8$) when they are almost transparent. Again, this strong dependence of the self-similar properties on the foliage transparency can be interpreted through light interception. When foliages are opaque ($\alpha_{\text{fol.}} = 0$), only the outer foliages intercept light, and architecture remodeling converges towards a crown and a tree structure of dimension $D = 2$. On the contrary, when foliages are fully transparent ($\alpha_{\text{fol.}} = 1$), one expects volume filling that correspond to $D = 3$. For intermediate transparencies, and for trees of finite size, intermediate fractal dimensions emerge. Taken together, our sensitivity analyses on MECHATREE and on the AMT model, as well as the present parametric analysis on the fittest species, show that our explanations of the empirical allometric laws are robust.

SUPPLEMENTARY DISCUSSION

Comparison between the WBE and AMT models

The West, Brown, and Enquist (WBE) model[1] was initially developed to investigate how the optimisation of the hydraulic performance affects the allometric structure of a vascular network. Later application of this model to plants[2], and subsequent related models[3,4], combined hydraulics with a mechanical assumption (generally elastic similarity). The application of the WBE model to plants has been very successful and the model has become a standard of plant ecology. It is therefore useful to compare our approach with the WBE model.

The WBE model should be compared with the AMT model, as only comparisons between models of similar types (in this case, static optimal models) are meaningful. To identify the similarities and differences between these two models, it is useful to derive their results from similar assumptions, with the same notations. Note that the present derivation of WBE allometric equations does not use the first principles outlined in the seminal WBE papers[1,2]. Note also that alternative derivations of the WBE equations are possible, based on different assumptions. Here, we derive the WBE allometric scalings from three assumptions:

i. Self-similarity of the tree skeleton;

ii. Volume filling;

iii. Pipe model or elastic similarity.

For the third assumption one has a choice: as already noted by Savage *et al.*[4], both the area conservation associated with the pipe model[5] (used in the original WBE model[1]) and the elastic similarity scaling law (used in later versions

of the WBE model[2]) yield similar relationships when combined with volume filling. As this work is centered on plant biomechanics we retain elastic similarity here.

Assumption (i) is shared the AMT model and the present derivation of the WBE results: tree skeletons are assumed to be self-similar structures (here self similarity is assumed for simplicity; it was found to emerge in the MECHATREE numerical model, or was derived from the principles of minimal hydraulic conductance and volume filling in the seminal derivation of the WBE model for plants[2]). Assumption (ii) is based on an analogy with arterial and bronchial networks, for which volume-filling makes sense because every part of the volume should be irrigated by arteries or bronchia. Assumption (iii), or elastic similarity, states that aspect ratio of branches has been selected such that the deflection of their tip Δ_k under self-weight is proportional to their length l_k for a given orientation[6], and, for upright branches, to provide an optimal resistance to elastic buckling[2–4]. It was found that these branches should follow the scaling law $l_k \propto d_k^{2/3}$.

First, self-similarity provides a relation between the branching and length ratio: $R_l = R_n^{1/D}$ (Assumption i). Then, elastic similarity provides a relation between the diameter ratio and the length ratio: $R_d = R_l^{3/2}$ (Assumption iii). To go further, one can write the allometric relation between path length and diameters[7]

$$\langle l \rangle_k = C \left(\frac{E}{g\tilde{\rho}_{\mathrm{wood}}} \right)^{\frac{1}{3}} d_k^{\frac{2}{3}}, \tag{1}$$

where E is the Young's modulus, g is the acceleration of gravity, $\tilde{\rho}_{\mathrm{wood}}$ is the density of green wood, and C is a dimensionless constant such that $C(E/g\tilde{\rho}_{\mathrm{wood}})^{1/3} \approx 25\,\mathrm{m}^{1/3}$[6,8]. This relation, where the path length can be related to R_l and k through (5), is the equivalent of equation (8) in the AMT model.

Now assuming that $D = 3$ (Assumption ii), $R_n = 2$, taking again $L = 10\,\mathrm{cm}$, $\rho_{\mathrm{wood}} = 613\,\mathrm{kg\,m^{-3}}$, and $m_{\mathrm{fol.}} = 1.8\,\mathrm{g}$, and using the same line of thought as above, we can derive the following allometric relations

$$H \approx 25\, d_{\mathrm{trunk}}^{0.67}, \tag{2}$$
$$M_L \approx 247\, d_{\mathrm{trunk}}^{2}, \tag{3}$$
$$M_L \approx 0.176\, M_S^{0.75}. \tag{4}$$

Note that usually, when the WBE model is presented[2–4], only the allometric exponents are given (Supplementary Table 1), and not the allometric constants. These relations are similar to what has been obtained in the AMT model (eqns. 13–15 above).

In the AMT model, there are also three assumptions, somewhat comparable to the above assumptions of the WBE model:

i. Self-similarity;

ii. Fractal dimension $D \approx 2.5$;

iii. Constant safety against wind-induced pruning.

The allometric scalings predicted by the WBE and AMT models are compared with empirical data found in the literature in Fig. 6 of the main text. This comparison shows that both modeling approaches give similar results in excellent agreement with the observations, both for the allometric exponents and for the allometric intercepts. The difference is that, in the AMT model, the three main assumptions are in fact emergent properties of the simulations of MECHATREE. Self-similarity with a fractal dimension $D \approx 2.5$ has been measured for the fittest species (Assumptions i and ii). It emerges from the competition for light for a foliage transparency of 0.5 (Section "Parametric analyses" in the main text and Supplementary Figs. 12h–i). The simulations have also shown that the safety factor against wind is almost constant (Assumption ii): $2.5 \lesssim S \lesssim 4$ (Supplementary Fig. 6c).

Growth strategies for the periphery species

The resource allocation strategy of each species is determined by its genome. In particular, the genome sets the neural coefficients of the two formal neural networks affecting the primary and secondary growth. The action of these

SUPPLEMENTARY TABLE 1. **Comparison of the allometric exponents in the WBE and in the AMT models.**

	β_H	β_N	β_{ML}
WBE model ($D = 3$)	$\frac{2}{3}$ (0.67)	$\frac{2D}{3}$ (2)	$\frac{D}{4}$ (0.75)
AMT model ($D = 2.5$)	$\frac{3}{D+1}$ (0.86)	$\frac{3D}{D+1}$ (2.14)	$\frac{3D}{2D+5}$ (0.75)

networks is represented in Supplementary Fig. 2 for the fittest species (the periphery species), and in Supplementary Fig. 3 for the other finalist species (the interior species).

The secondary-growth neural network has two inputs: the number of foliages feeding a segment (i.e. the number of foliages situated above in the hierarchy), and $\sigma_{\max}/\sigma_0$ the relative stress perceived by the segment. The stress $\sigma_{\max}$ is calculated in each segment as the maximum bending stress experienced for winds of constant speed U_0 with orientations separated by 45° along the azimuth (see Methods in the main text). The speed U_0 is chosen to give a Cauchy number $C_Y = \rho U_0^2/\sigma_0 = 2 \times 10^{-5}$, where ρ is the air density and σ_0 is the strength of wood.

The secondary-growth neural network has a single output: the safety factor S. The safety factor is used to calculate the biomass requested by each segment: the volume requested is aimed at reaching a volume $V = SV_{\text{fract.}}$, with $V_{\text{fract.}}$ the volume giving $\sigma_{\max} = \sigma_0$. In other words, segments request an increase of their diameter to reach a stress $\sigma_{\max} = \sigma_0/S^{3/2}$, where the 3/2 exponent comes from the fact that the volume scales as d^2 and the maximum stress as d^3. It should be noted however that a constant safety factor does not imply that all segments have the same probability of fracture. This is due to the form of the Weibull distribution used to model this probability, which includes a volume effect, as seen in eq. (3) in the main text. Constant probability would be achieved for $S \propto d^{4/3m}$, with $m = 10$ the Weibull modulus. Because of area conservation, the segment diameter roughly increases as $N^{1/2}$, with N the number of foliages irrigating the segment. Constant probability of fracture would thus happen for $S \propto N^{2/3m}$.

In Supplementary Figs. 2a–b, the safety factor "computed" by a segment through the secondary-growth neural network is plotted for the periphery species. It shows that S takes a typical value of 3, which gives a probability of fracture for a wind speed U_0 of the order of $P \approx 1 - \exp(-10S^{3m/2}) \approx 7 \times 10^{-7}$ (eq. (3) in the main text). However for large wind speeds U, this typical probability becomes $P \approx 1 - \exp(-10\, S^{3m/2}(U/U_0)^{2m})$. Taking $U/U_0 = 1.5$, which happens every 100 yrs on average[9], the probability of fracture is of the order of $P \approx 2 \times 10^{-3}$. Since large trees are composed of thousands of segments, this probability is not negligible and during its lifetime a tree will have some branches pruned by the wind.

In Supplementary Fig. 2a, the safety factor is plotted for typical values of the relative stress $\sigma_{\max}/\sigma_0 = 0.15$ and 0.20. It shows that when $N \lesssim 40$, S is roughly constant, meaning that branches of moderate diameter are more likely to break than small diameter ones. For $8 \lesssim N \lesssim 200$, the slope of the safety factor (in this log-log representation) is approximately equal to $2/3m$, which means that large branches have an constant probability to break. For very large diameters ($N \gtrsim 400$), S is roughly constant again, which means that the trunk may be somewhat fragile to very strong wind.

The primary-growth neural network takes two inputs: the total number of foliages $N_{\text{tot.}}$ in the tree and the relative biomass in the reserve, which is $V_{\text{reserve}}/(N_{\text{tot.}}V_0)$, with V_{reserve} the volume in the reserve. This neural network has three outputs: the percentage of biomass allocated to grow seeds, the percentage of biomass allocated to grow new segments, and a photosensitivity parameter $0 \leq p \leq 1$. The value of p is used to calculate the positions of the new segments and seeds. For $p = 0$, new segments are added at random foliage locations, and for $p = 1$, the n new segments are added at the locations of the n most lit foliages. For intermediate values, the pool of foliages among which n locations are randomly selected is composed of the $n + (1-p)(N_{\text{tot.}} - n)$ most lit foliages. The rule to choose seed locations is the same as for new segments.

Supplementary Figs. 2c–e show the outputs of the primary-growth neural network for the fittest species. The strategy used by this species is the following: for small trees, or when the reserve is important, all the biomass is allocated to grow new segments. However when the reserve is relatively low, most of the biomass is allocated to grow seeds. The photosensitivity parameter for this species is always larger than 0.1. It reaches a maximum when the tree is large and the relative biomass is low.

Growth strategies for the interior species and other species

For comparison purposes, the outputs of the primary- and secondary-growth neural networks have been plotted for the other finalist of the tournament in Supplementary Fig. 3. We also plotted the self-similar characteristics of a tree of the interior species growing without competitor (Supplementary Fig. 10). Most characteristics are similar to those of the fittest species. For instance, the typical safety factor is still between 2.5 and 4 and the overall tendency as the number of foliages increases is the same (Supplementary Figs. 2a and 3a). Self-similar ratios are also similar for both species, and the fractal dimension is still approximately $D = 2.5$ (Supplementary Fig. 10b).

However, the allocation of biomass is arbitrated somehow differently between seeds and new segments. For the interior species, the biomass in the reserve is always fully allocated to grow seeds or new segments; there is no stock from year to the other contrarily to what the periphery species does. Another interesting feature is that this arbitrage is almost independent on the number of foliages. Therefore, the strategy is roughly the same for small, medium, or large trees. For the photosensitivity parameter p, the main difference with the periphery species is that, here, p reaches its maximal possible value $p = 1$ as soon as the relative biomass volume is over 0.5, which means that the

trees of the interior species will mainly grow (by the addition of new segments) where there is a maximum of light.

The two finalist species can coexist on large islands by living in two distinct ecological niches: the periphery and the interior of the island. At present, it is not entirely clear how the differences in allocation strategies are related to these two niches. It is likely linked to the photosensitivity, the management of reserve biomass, or the relative fragility of the interior species (wind-induced pruning are more frequent for same wind conditions as evidenced by the discontinuities of the fractal dimension in Supplementary Fig. 10c), but further studies are needed to clarify this point.

The differences between Supplementary Figs. 2 and 3 show that there may be several good resource allocation strategies to grow in a competing environment. To evaluate the space of "acceptable" growth strategies, the branching angles and the outputs of the neural networks have been compared for all the winning species of the 32 first-round forests (Supplementary Fig. 6). Looking at the branching angles (Supplementary Fig. 6a–b), it shows that all species lie around the line $\theta_1 - \theta_2 \approx 90^\circ$, meaning that children segments tend to form a normal angle. The angle γ that is used to obtain a non planar geometry (which can be interpreted as a phyllotaxy angle) is much less constrained. It takes any values except around 0 (or 360°), and 180°, for which the geometry would indeed be planar.

For all species, the safety factor has similar values around 3 (Supplementary Fig. 6c). This suggests that this corresponds to an optimal tradeoff between mechanical resistance to wind and biomass minimisation. This optimum likely takes into account the distribution of wind speeds along time: for $S \approx 3$ and for average wind speeds, trees statistically would not break, as discussed above, but for extreme wind events, some branches will eventually be pruned.

Supplementary Figures. 6c and 6d also show that the photosensitivity parameter takes values between 0.5 and 1 for almost all species. Growing new segments on the most lit branches thus seems to give an evolutionary advantage in the simplified environment of the simulations. The primary-growth neural network further show that the biomass contained in the reserve is almost always entirely allocated between seeds and new segments (with the notable exception of the periphery species though). The percentage allocated to new segments is roughly between 35 and 90%, although it should be noted that this figure only shows the allocation for "typical" trees with 20 foliages and a relative reserve of 0.2.

Note on the tapering law

McMahon & Kronauer[6] proposed that the tapering exponent should be $\beta = 2/3$, with the argument of elastic similarity. This exponent reasonably fits the data for moderate and large Strahler orders in the present simulations (Fig. 5d and Supplementary Fig. 8d). It should be pointed out however that elastic similarity is not implemented in the present model. Here, this scaling results mainly from the growth strategy implemented by the tree to withstand wind-induced loads.

The AMT model actually predicts an exponent $\beta = \frac{D+1}{3}$, which is equal to 1.17 for $D = 2.5$ (see above). Yet, it can be seen that the highest Strahler order branches seems to follow a scaling with a lower exponent (Fig. 5d and Supplementary Fig. 8d). Several explanations can be put forward to explain this apparent discrepancy. First, the safety factor increases slightly with the Strahler orders (Supplementary Fig. 2a), resulting in a relative reinforcement of large order branches. Second, for the branches at the base of the structure, the bending moment due to wind-induced loads on the branches themselves cannot be neglected anymore when compared to the wind loads on the foliages (this is because the sail area of the foliages is of same order as the sail area of the trunk). Third, the length of the trunk is lower than predicted by a geometric progression in general (e.g., in Supplementary Fig. 8b, the 7th order branch length is well below the regression line). The first two arguments may explain why large Strahler order branches may have a larger diameter than predicted, and the last argument explains why their length could be smaller. Combining these effects thus tend to lower the allometric exponent β for the largest branches.

SUPPLEMENTARY TABLE 2. **Sensitivities of the allometric constants on model parameters.** This table reports sensitivities $s_{a,b}$, which quantify how each allometric constants (the exponents β_X or the intercepts α_X) depend on the values of the model parameters. These sensitivities are estimated with the data plotted in Supplementary Fig. 11. The stars indicate whether the 95%, 99%, or 99.9% confidence interval entirely lies above or below 0 (resp. 1, 2, or 3 stars).

Parameter	β_H	β_N	β_B	β_{ML}	α_H	α_N	α_B	α_{ML}
C_Y	0.030	-0.005	-0.019^{*}	0.002	-0.327^{**}	-0.522^{**}	-0.262^{***}	-0.344^{*}
e	0.270^{***}	-0.025	-0.025	-0.027	0.130^{*}	0.462^{***}	0.114^{**}	0.342^{***}
$V_{\text{prod.}}$	-0.221^{***}	-0.091^{*}	0.053^{*}	-0.091^{*}	0.054^{*}	-0.495^{***}	-0.049	-0.197^{***}
$\alpha_{\text{fol.}}$	-0.144^{*}	0.047	-0.017^{*}	0.073^{*}	-0.289^{***}	0.105	-0.176^{**}	0.124^{*}

SUPPLEMENTARY TABLE 3. **Theoretical sensitivities of the allometric constants based on the AMT model.** These theoretical values are predicted by the AMT model (see section "Principle of the AMT model" in Methods), to be compared to the values measured in the simulations (Supplementary Table 2). There is no dependence on the parameters not shown.

Parameter	β_H	β_N	β_B	β_{ML}	α_H	α_N	α_B	α_{ML}
C_Y	0	0	0	0	$-\frac{2}{7}$ (-0.143)	$-\frac{5}{7}$ (-0.714)	$-\frac{20}{21}$ (-0.952)	0
$\alpha_{\text{fol.}}$	$-\frac{1}{7}$ (-0.143)	$\frac{2}{35}$ (0.057)	$-\frac{3}{70}$ (-0.043)	$\frac{1}{10}$ (0.1)	-0.442	0.442	-0.442	0

SUPPLEMENTARY TABLE 4. **Sensitivities of safety, fractal dimension, and tree size to model parameters.** Similar to Supplementary Table 2, but for S, D, and $\log_{10} N_{\text{seg.}}$ (data plotted in Supplementary Fig. 11, last column).

Parameter	S	D	$\log_{10} N_{\text{seg.}}$
C_Y	-0.236^{**}	0.125^{**}	-0.193^{***}
e	-0.155^{**}	0.087^{**}	-0.446^{***}
$V_{\text{prod.}}$	0.110^{*}	-0.153^{***}	0.404^{***}
$\alpha_{\text{fol.}}$	0.122^{*}	0.126^{**}	0.236^{***}

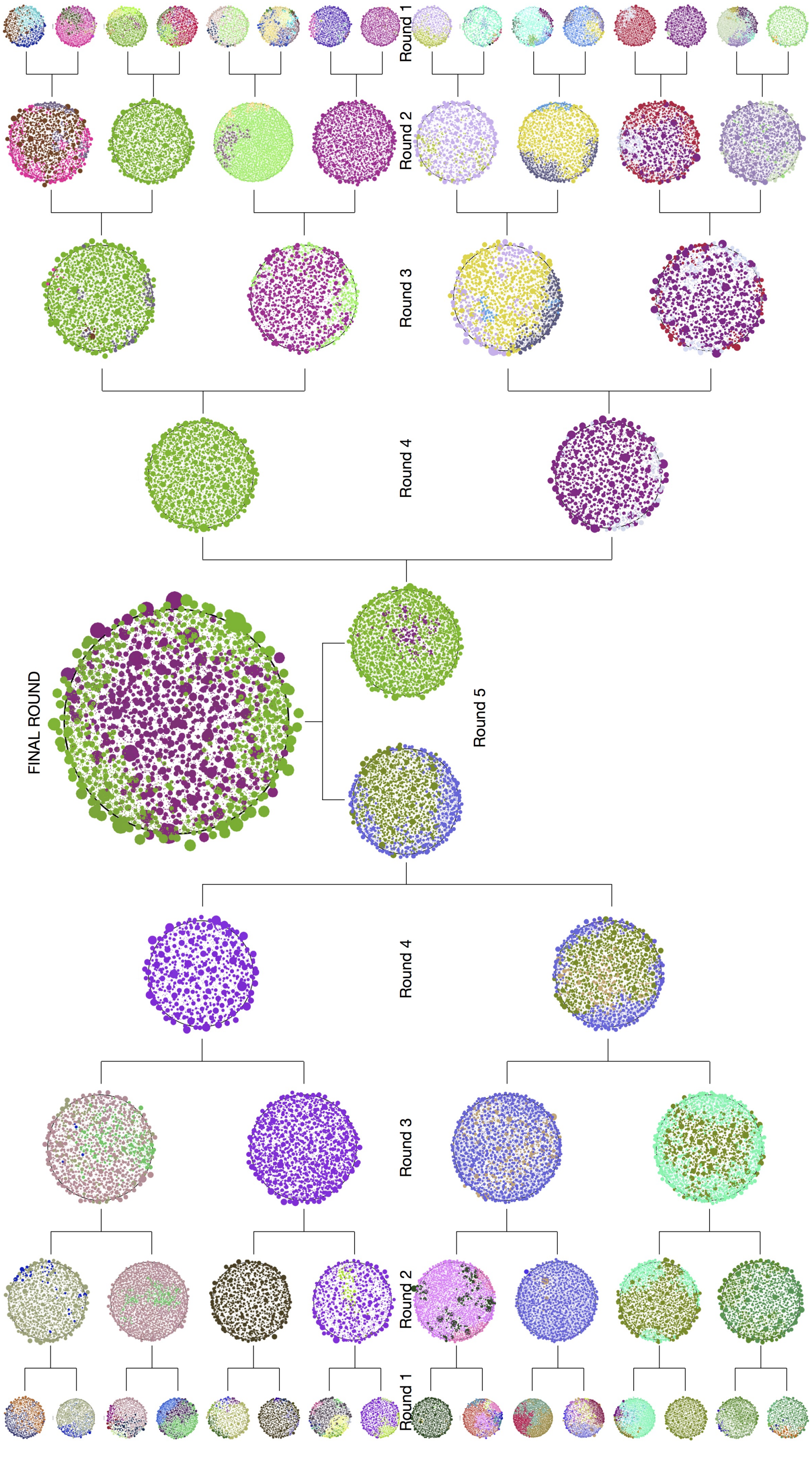

SUPPLEMENTARY FIGURE 1. **Tournament to identify fittest species.** Illustration with the same conventions as in Fig. 2 (main text) of the species left on the island after each round. It can be seen that at the end of the Final round, two species remain: one living on the periphery (green), one living in the interior of the island (purple).

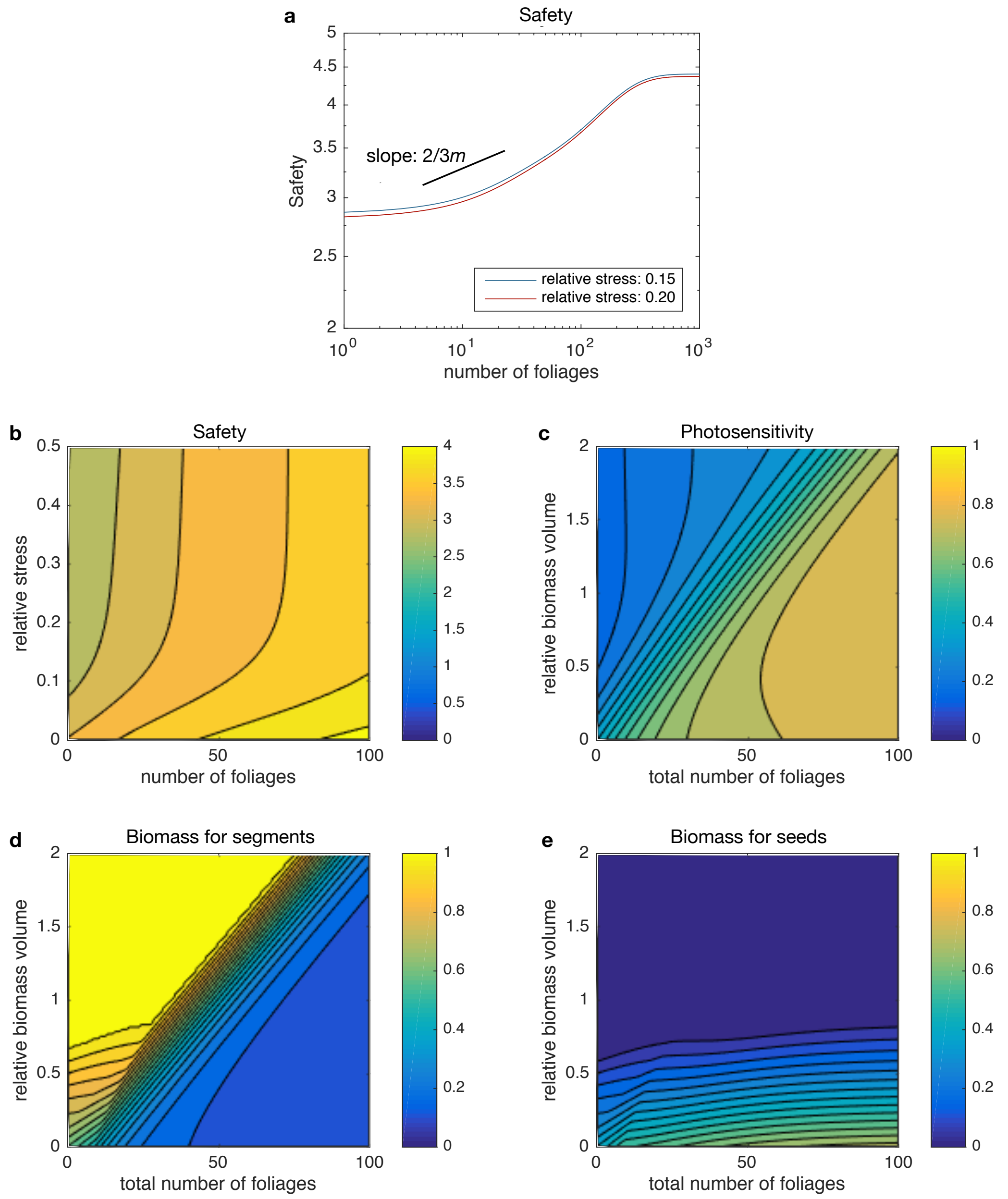

SUPPLEMENTARY FIGURE 2. **Resource allocation strategy of the periphery species (the fittest species).** (**a**) Safety coefficient computed by the secondary-growth neural network for two typical values of the relative stress: 0.15 and 0.20. (**b**) Result of the secondary-growth neural network: Safety coefficient as a function of the relative stress in the segment and the number of foliages below in the hierarchy. The graph in (a) is a horizontal cut of the present contour plot. (**c**) Photosensitivity parameter as a function of the total number of foliages in the tree and the relative biomass in the reserve. (**d**) Percentage of the reserve biomass allocated to the growth of new segments. (**e**) Percentage of the reserve biomass allocated to the growth of seeds. (c, d, and e show the 3 outputs of the primary-growth neural network as a function of the 2 inputs.)

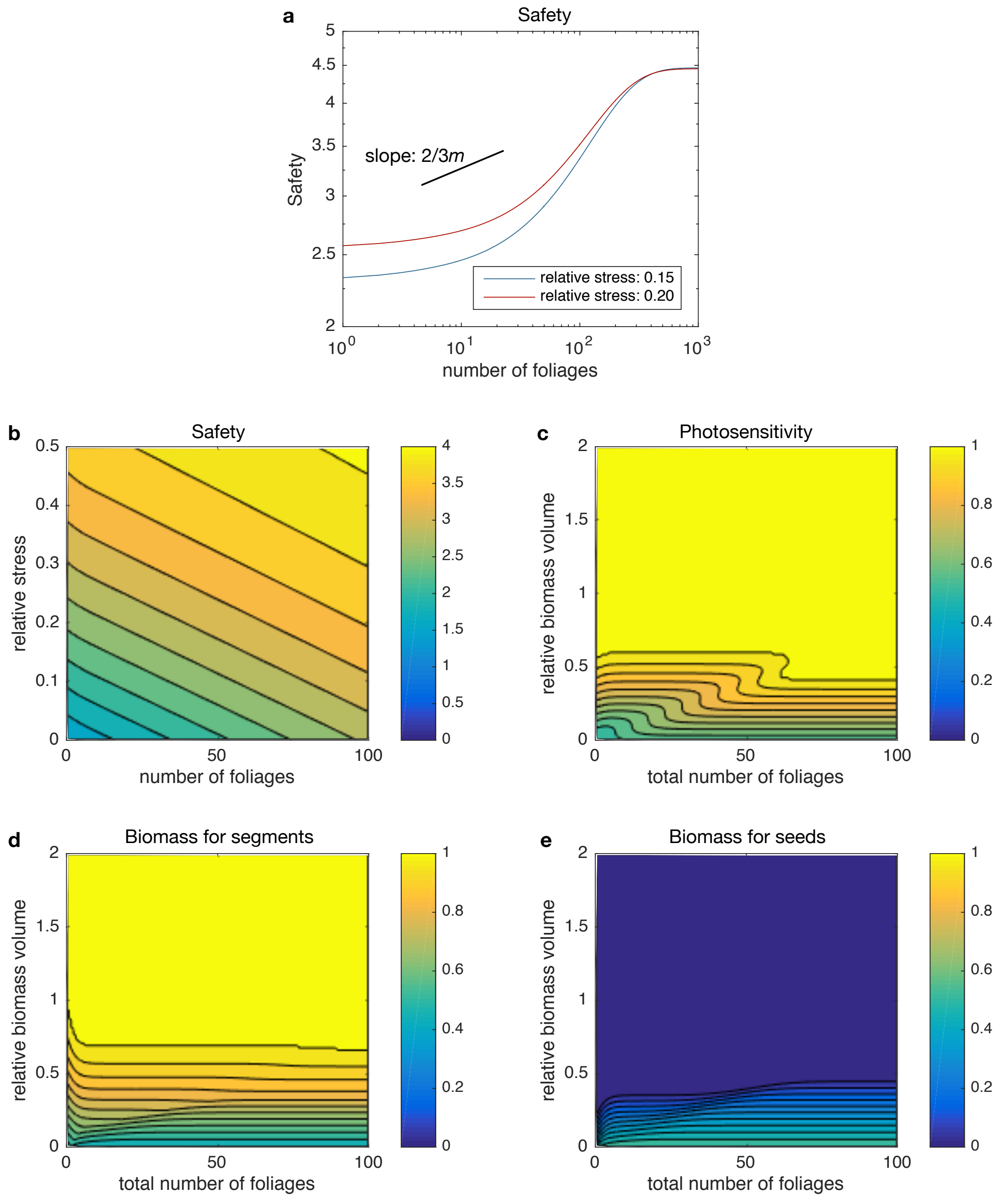

SUPPLEMENTARY FIGURE 3. **Resource allocation strategy of the interior species.** Same as Supplementary Fig. 2 but for the species of the other finalist in the tournament: the interior species.

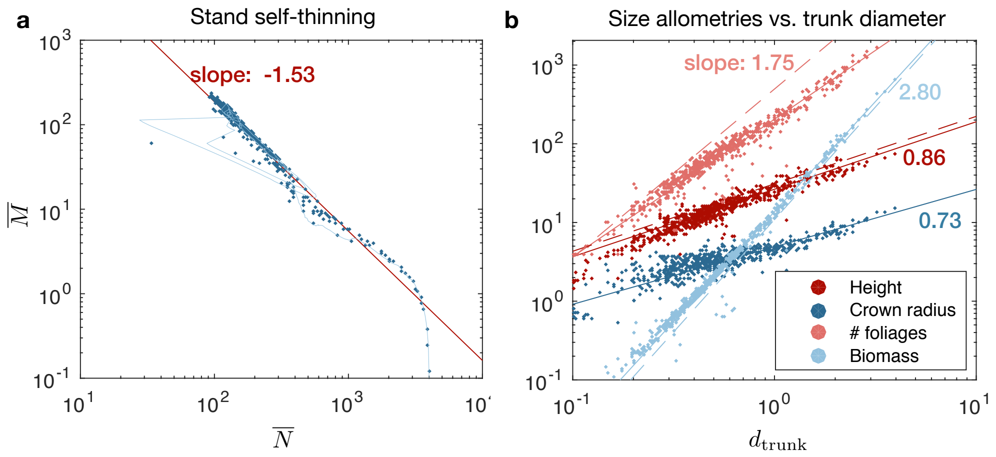

SUPPLEMENTARY FIGURE 4. **Allometric scalings for forests with the two finalist species (periphery and interior species).** (**a**) Same as Fig. 3a in main text for 16 forests planted with the two finalist species after 3,000 yrs of simulation. (**b**) Same as Fig. 3b for the same two-species forests as in (a). Dashed lines show predicted allometric laws (see section "Comparisons with virtual forests and empirical data" in Methods).

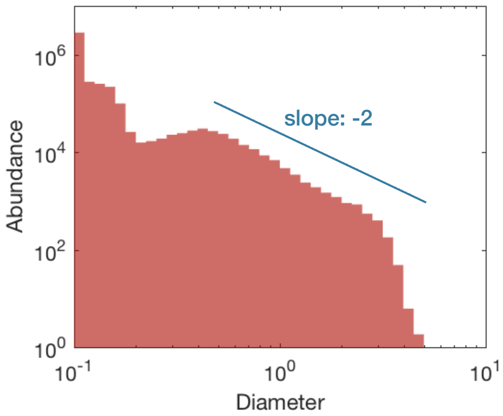

SUPPLEMENTARY FIGURE 5. **Size distribution in forests with the two finalist species (periphery and interior species).** This plot shows the distribution of diameters in the 16 forests planted with the finalist species (same forests as for Supplementary Fig. 4). For small diameters, the distribution is biased by the large amount of young trees, which follow the second death rule (the tree dies if it does not have at least 10 segments after 6 yrs). For larger diameter ($d_{\mathrm{trunk}} > 0.5$), the distribution follows the scales as d_{trunk}^{-2}.

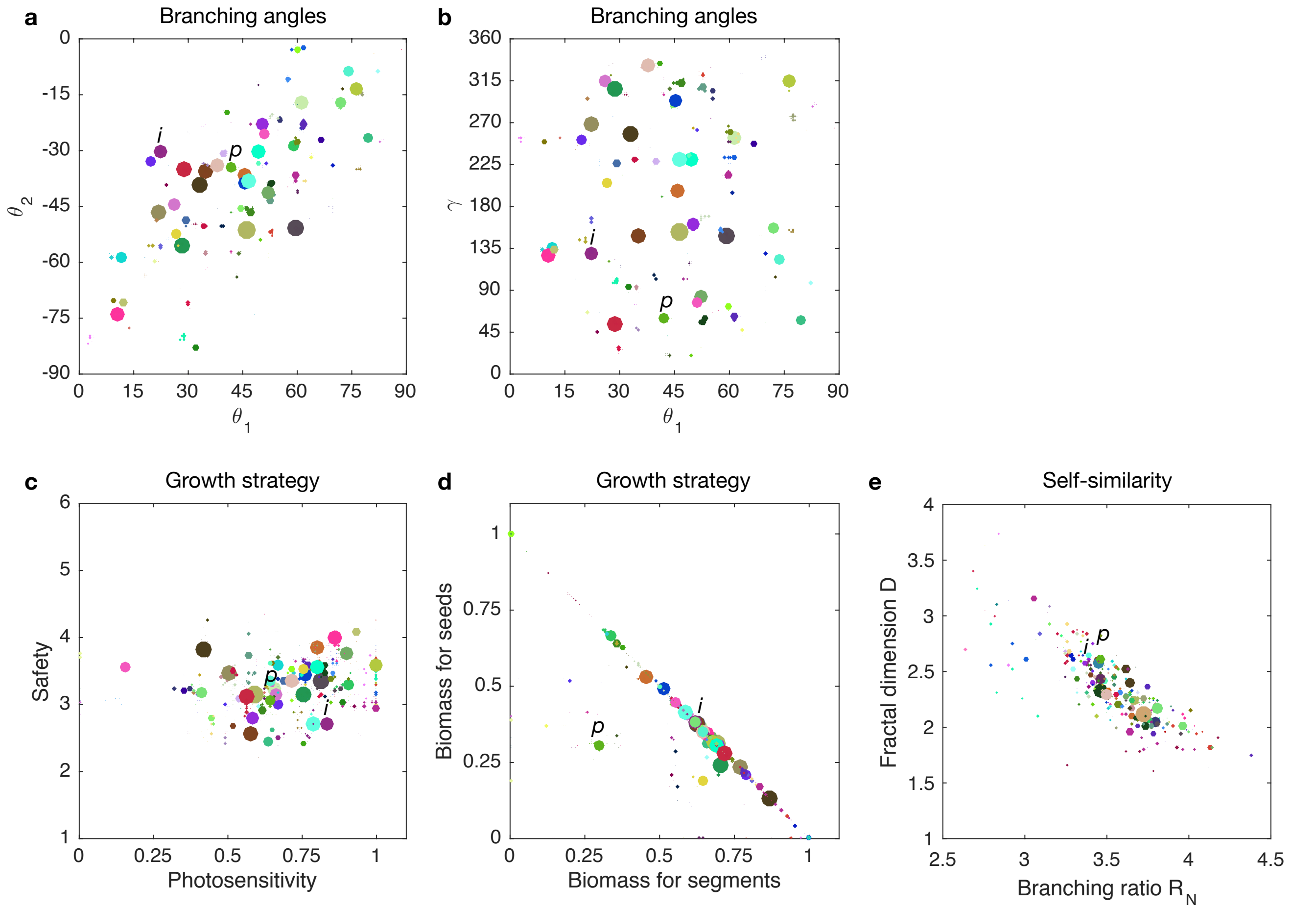

SUPPLEMENTARY FIGURE 6. **Growth strategies of the 1st-round winners.** (**a–d**) Each point represents a tree in one of the 32 islands of the first round (see Supplementary Fig. 1). The ancestor of the periphery species is marked with a 'p', and the ancestor of the interior species with an 'i'. The output of the secondary-growth neural networks is given for a typical segment irrigated by 10 foliages and perceiving a relative stress of 0.2. The outputs of the primary-growth neural network are given for a typical tree with 20 foliages and a relative reserve biomass volume of 0.2 (see Supplementary Figs. 2 and 3 and section "Growth strategies for the interior species and other species" in Methods). (**e**) Each genome appearing in figures (a–d) is used to initialise a 200-yr simulation of growth without competitor (similar to Supplementary Fig. 12). From these simulations, the branching ratio and the fractal dimension are calculated: $R_N = 3.50 \pm 0.32$ and $D = 2.44 \pm 0.65$ (mean and standard deviation).

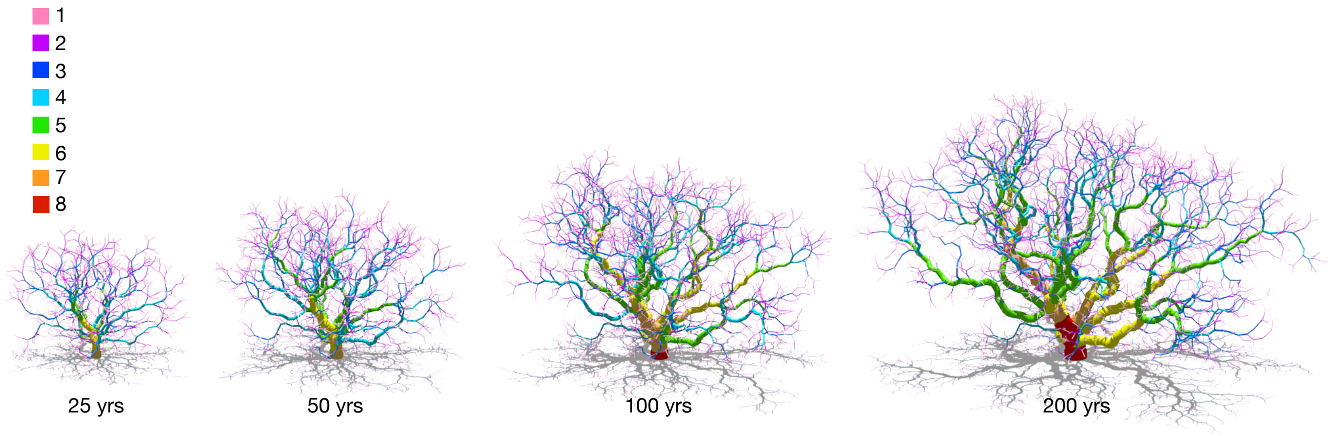

SUPPLEMENTARY FIGURE 7. **Growth of a tree without neighbours.** Growth of the fittest species, the periphery species (same as in Fig. 4, main text). However, because of the absence of competing neighbours, its shape is qualitatively different. The same colour code is used for the Strahler rank of branches (Supplementary Movie 3).

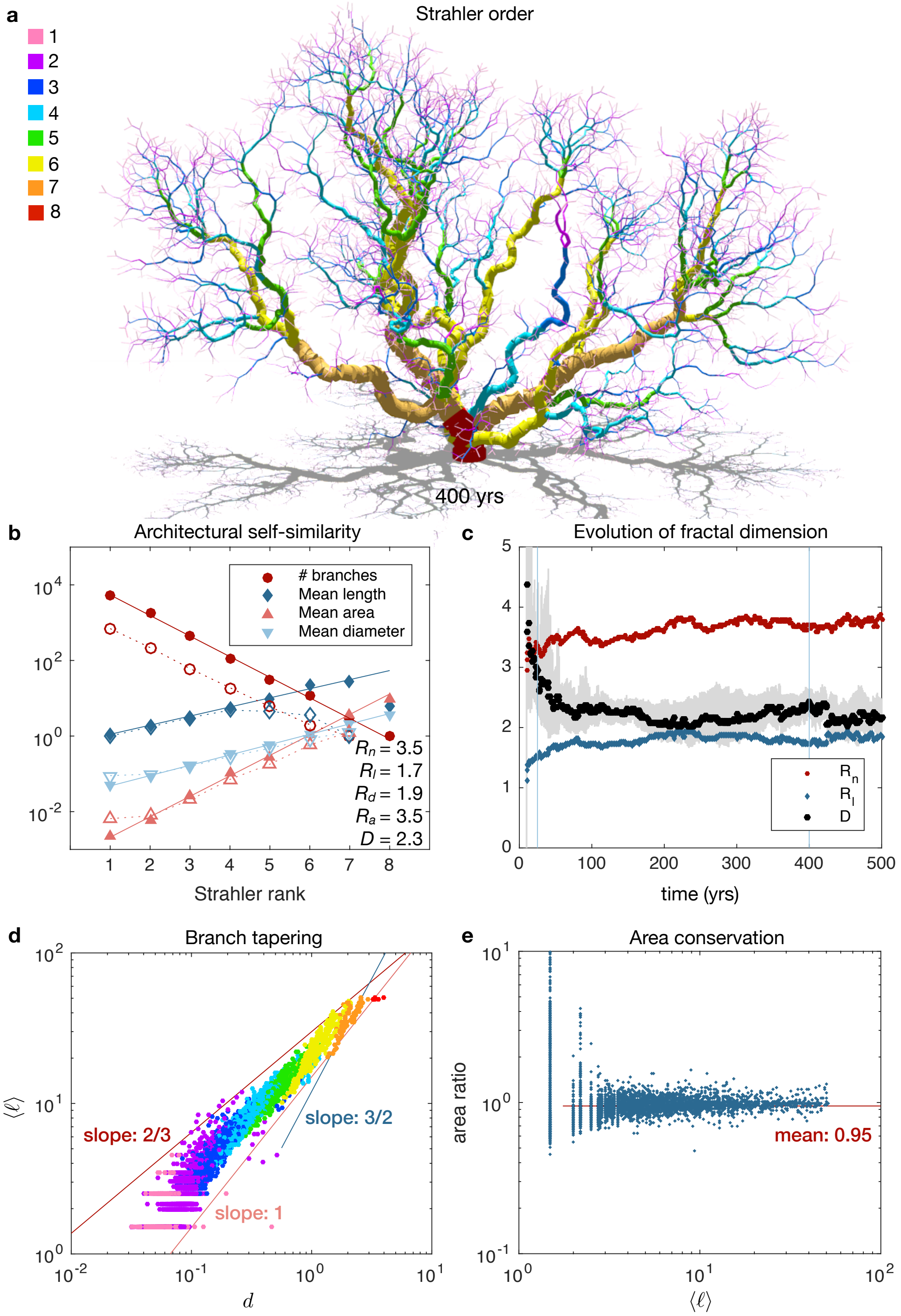

SUPPLEMENTARY FIGURE 8. **Self-similarity of a tree without neighbours.** Same as Fig. 5 (main text) but for a tree growing without any competitors (same tree as depicted in Supplementary Fig. 7, but after 400 years).

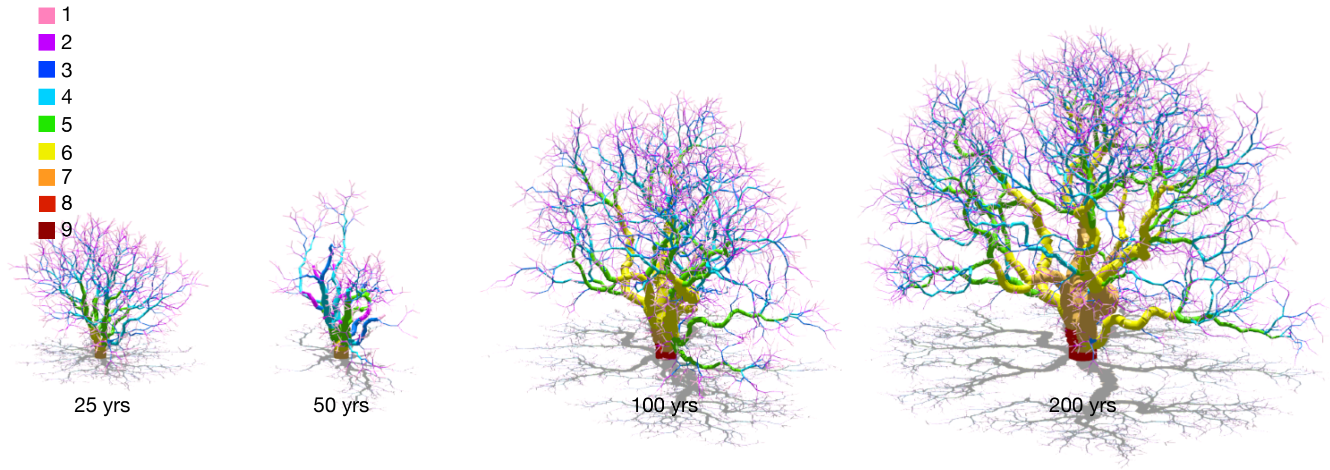

SUPPLEMENTARY FIGURE 9. **Growth of a tree from the interior species without neighbours.** Same as Fig. 7, but for the other finalist species (the interior species).

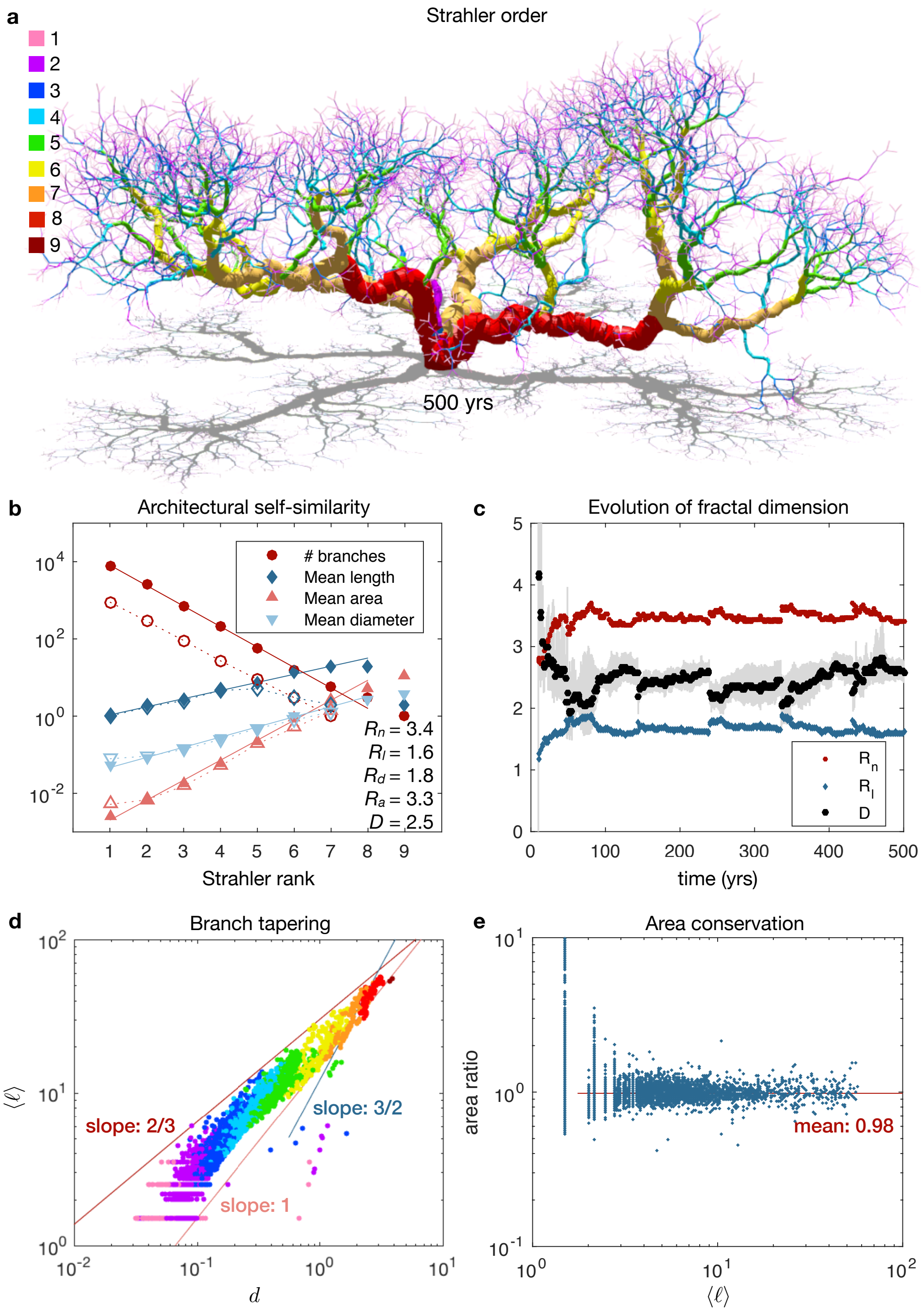

SUPPLEMENTARY FIGURE 10. **Self-similarity of a tree from the interior species without neighbours.** Same as Fig. 8, but for the other finalist species (the interior species).

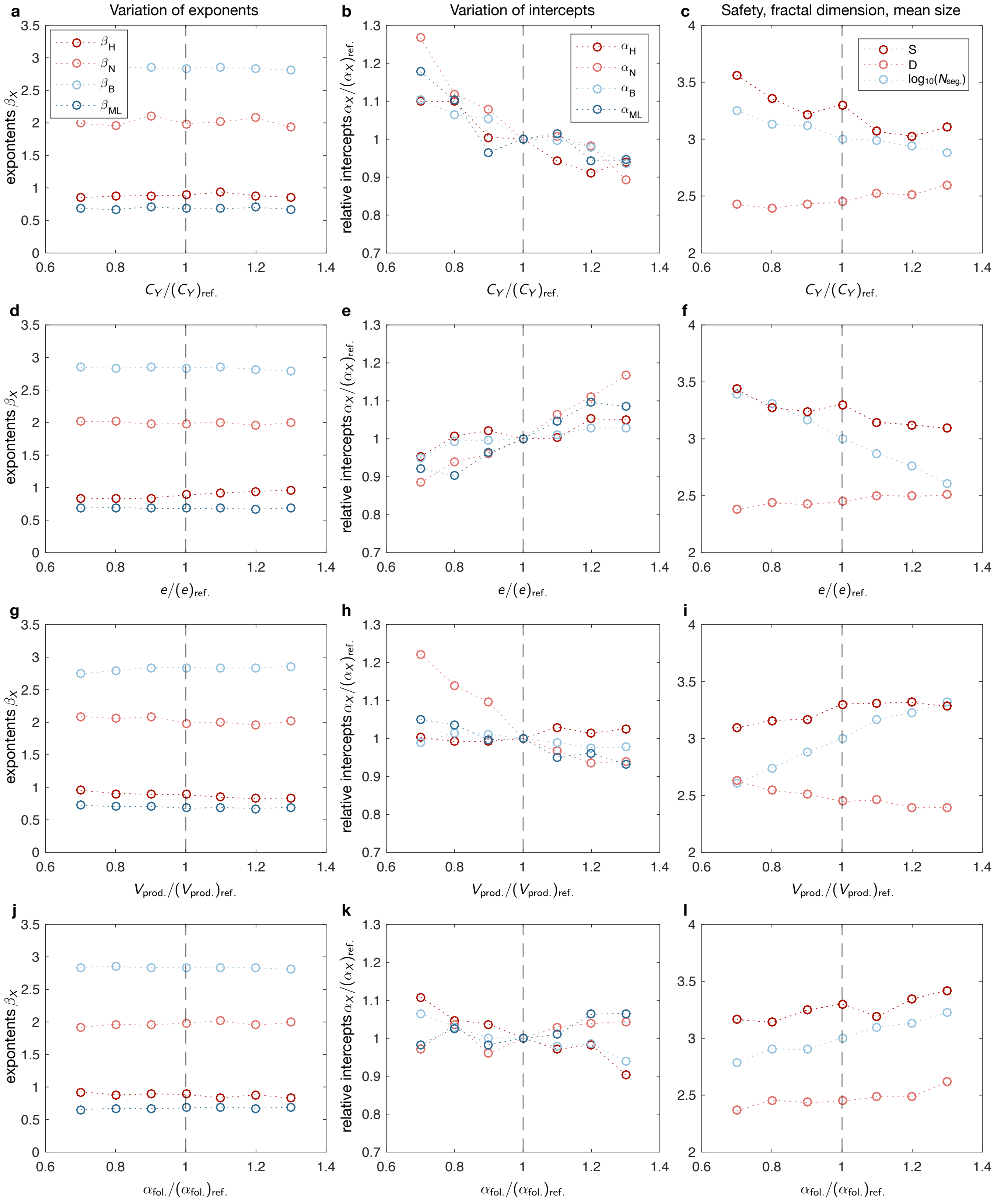

SUPPLEMENTARY FIGURE 11. **Sensitivity analysis on the allometric laws.** (**a**) Variation of the allometric exponents β_H, β_N, β_B, and β_{ML}, when the Cauchy number is varied around its reference value $(C_Y)_{\mathrm{ref.}} = 2\times10^{-5}$. Each point corresponds to 16 forests ran during 3,000 yrs and the allometric exponents are determined through a standard major axis regression (see Fig. 3b in main text). (**b**) Same as (a) but for the relative intercepts of the allometric laws α_X. (**c**) For the same forests as the ones used in (a,b), we plot the average safety factor S for a typical segment (irrigated by 10 foliages and sensing a relative stress of 0.2, see Supplementary Fig. 6c), the logarithm of the average number of segments $N_{\mathrm{seg.}}$ (excluding small trees for which $H < 10L$), and the average fractal dimension D (calculated from 200 yr-long simulations of trees without competitors, see Supplementary Fig. 6e). (**d–i**) Each line corresponds to the same plots, but for a variation of another model parameter: the maintenance thickness e (d–f), the volume produced by foliages $V_{\mathrm{prod.}}$ (g–i), and the foliage transparency $\alpha_{\mathrm{fol.}}$ (j–l).

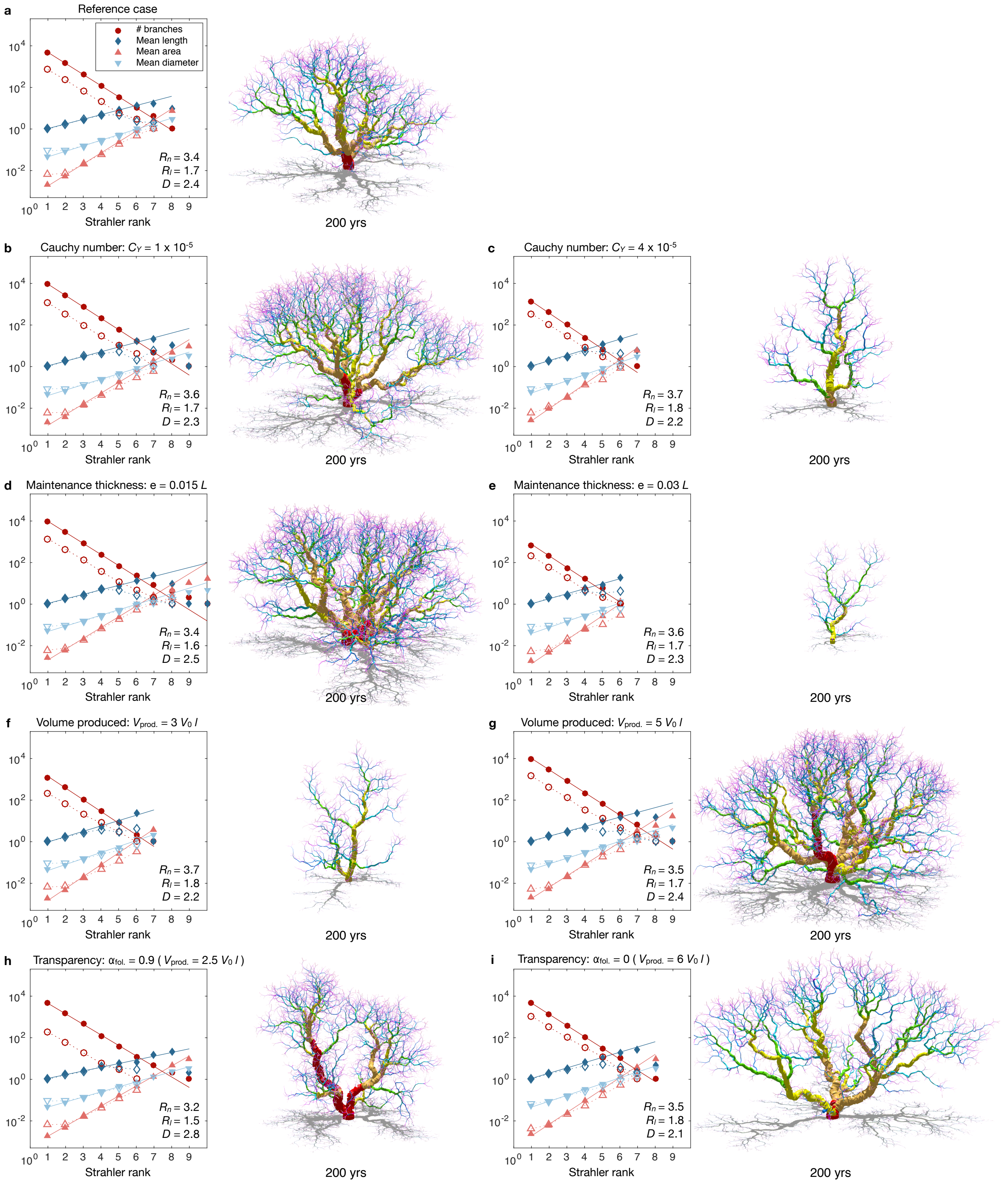

SUPPLEMENTARY FIGURE 12. **Parametric analyses.** (**a**) Reference case for Cauchy number $C_Y = 2\times10^{-5}$, maintenance thickness $e = 0.02L$, biomass volume produced $V_{\mathrm{prod.}} = 4V_0 l$, and optical transparency of foliages $\alpha_{\mathrm{fol.}} = 0.5$. The left graph shows the tree self-similarity (dashed lines and open symbols for the 25-year-old tree; solid lines and filled symbol for the 200-year-old tree). This graph is similar to Supplementary Fig. 8b (the only differences come from the stochasticity of wind, fracture, and branching angle). (**b–g**) Similar simulations but with one of the parameter changed compared to the reference case: Cauchy number (b, c); maintenance thickness (d, e), and biomass volume produced (f, g). (**h–i**) Simulations for a different transparency; the volume produced has also been modified such that the 200-year-old tree has approximately the same number of branches as in the reference case.

SUPPLEMENTARY REFERENCES

[1] Geoffrey B West, James H Brown, and Brian J Enquist, "A general model for the origin of allometric scaling laws in biology," Science **276**, 122–126 (1997).

[2] G.B. West, J.H. Brown, and B.J. Enquist, "A general model for the structure and allometry of plant vascular systems," Nature **400**, 664–667 (1999).

[3] Brian J Enquist and Karl J Niklas, "Global allocation rules for patterns of biomass partitioning in seed plants," Science **295**, 1517–1520 (2002).

[4] VM Savage, LP Bentley, BJ Enquist, JS Sperry, DD Smith, PB Reich, and EI von Allmen, "Hydraulic trade-offs and space filling enable better predictions of vascular structure and function in plants," Proc. Nat. Acad. Sc. USA **107**, 22722–22727 (2010).

[5] K. Shinozaki, K. Yoda, K. Hozumi, and T. Kira, "A quantitative analysis of plant form–the pipe model theory. I. basic analyses," Jpn. J. Ecol. **14**, 97–105 (1964).

[6] T. A. McMahon and R. E. Kronauer, "Tree structures: Deducing the principle of mechanical design," J. Theor. Biol. **59**, 443–466 (1976).

[7] Karl J Niklas, "Interspecific allometries of critical buckling height and actual plant height," Am. J. Bot. **81**, 1275–1279 (1994).

[8] Gaëlle Jaouen, Tancrède Alméras, Catherine Coutand, and Meriem Fournier, "How to determine sapling buckling risk with only a few measurements," Am. J. Bot. **94**, 1583–1593 (2007).

[9] I D Morton, J Bowers, and G Mould, "Estimating return period wave heights and wind speeds using a seasonal point process model," Coast. Eng. **31**, 305–326 (1997).
