# Supplementary material for: Wind loads and competition for light sculpt trees into self-similar structures
Source: Nat Commun. 2017 Oct 18;8:1014. doi: 10.1038/s41467-017-00995-6 (PMC5715076; doi:10.1038/s41467-017-00995-6)
Supplement: Supplementary file 3 — Description of Additional Supplementary Files [file 41467_2017_995_MOESM3_ESM.pdf]

File Name: Supplementary Movie 1
Description: **Example of the growth and evolution of a virtual forest over 10,000 yrs.** This movie corresponds to Fig. 2.

File Name: Supplementary Movie 2
Description: **Three-dimensional growth of the Final round forest.** This movie corresponds to Fig. 4.

File Name: Supplementary Movie 3
Description: **Growth of a tree without neighbouring trees.** This movie corresponds to Supplementary Figs. 7 and 8.
